# Ontogenesis of the asymmetric parapineal organ in the zebrafish epithalamus

**DOI:** 10.3389/fcell.2022.999265

**Published:** 2022-12-09

**Authors:** Karina Palma, Iskra A. Signore, Margarita M. Meynard, Jazmin Ibarra, Lorena Armijo-Weingart, Marcos Cayuleo, Steffen Härtel, Miguel L. Concha

**Affiliations:** ^1^ Integrative Biology Program, Institute of Biomedical Sciences, Faculty of Medicine, University of Chile, Santiago, Chile; ^2^ Biomedical Neuroscience Institute, Santiago, Chile; ^3^ Center for Geroscience, Brain Health and Metabolism, Santiago, Chile; ^4^ Department of Physiology, University of Concepcion, Concepcion, Chile; ^5^ National Center for Health Information Systems (CENS), Santiago, Chile

**Keywords:** parapineal organ, epithalamus, habenula, ontogeny, zebrafish, neurochemistry, photoreceptor, ultrastructure

## Abstract

The parapineal organ is a midline-derived epithalamic structure that in zebrafish adopts a left-sided position at embryonic stages to promote the development of left-right asymmetries in the habenular nuclei. Despite extensive knowledge about its embryonic and larval development, it is still unknown whether the parapineal organ and its profuse larval connectivity with the left habenula are present in the adult brain or whether, as assumed from historical conceptions, this organ degenerates during ontogeny. This paper addresses this question by performing an ontogenetic analysis using an integrative morphological, ultrastructural and neurochemical approach. We find that the parapineal organ is lost as a morphological entity during ontogeny, while parapineal cells are incorporated into the posterior wall of the adult left dorsal habenular nucleus as small clusters or as single cells. Despite this integration, parapineal cells retain their structural, neurochemical and connective features, establishing a reciprocal synaptic connection with the more dorsal habenular neuropil. Furthermore, we describe the ultrastructure of parapineal cells using transmission electron microscopy and report immunoreactivity in parapineal cells with antibodies against substance P, tachykinin, serotonin and the photoreceptor markers arrestin3a and rod opsin. Our findings suggest that parapineal cells form an integral part of a neural circuit associated with the left habenula, possibly acting as local modulators of the circuit. We argue that the incorporation of parapineal cells into the habenula may be part of an evolutionarily relevant developmental mechanism underlying the presence/absence of the parapineal organ in teleosts, and perhaps in a broader sense in vertebrates.

## Introduction

The epithalamus is a region of the vertebrate brain comprising the habenulae and pineal complex. Left and right habenulae are a phylogenetically conserved bilateral nuclear complex that functions as a relay station connecting the limbic and striatal regions of the forebrain with the ventral midbrain and hindbrain (Bianco and Wilson, 2009; Aizawa et al., 2011). In contrast, the pineal complex consists of the pineal and parapineal organs, two structures that arise from evaginations located in the midline of the diencephalic roof plate (Oksche, 1965; Concha and Wilson, 2001) and which have changed markedly during evolution. For example, the pineal organ of mammals is a neuroendocrine gland indirectly regulated by the light-dark cycle, whilst in many groups of vertebrates it is a direct photosensory structure (Korf, 1999; Ekström and Meissl, 2003). The parapineal organ, on the other hand, has only been described in a subset of vertebrate species, including lampreys, the bowfin, the coelacanth, some teleosts, and lizards (where it is known as the parietal eye), and appears to be absent in hagfishes, cartilaginous fishes, amphibians, birds and mammals (Borg et al., 1983; Tsuneki, 1986; Concha and Wilson, 2001). It has been proposed that early vertebrates probably had both pineal and parapineal organs with direct photosensitivity, whereas present-day vertebrates with a single element in their pineal complex would have lost the parapineal organ during evolution (Concha and Wilson, 2001; Ekström and Meissl, 2003).

The parapineal organ of the teleost zebrafish (*Danio rerio*) has been extensively studied since its epithalamus has become the preferred model to investigate genetic and morphogenetic mechanisms that establish brain asymmetry in vertebrates. During development, the parapineal organ adopts a left-sided position promoting the development of conspicuous left-right habenular asymmetries at both structural and functional levels. Those asymmetries are lost when the parapineal organ is absent due to mutations (Snelson et al., 2008a; Regan et al., 2009; Clanton et al., 2013) or physical ablation (Concha et al., 2003; Gamse et al., 2003; Bianco et al., 2008; Lekk et al., 2019). Furthermore, ectopic transplantation of parapineal cells into the embryonic right habenula induces the expression of genetic markers usually expressed in the left, indicating that the parapineal is not only required but is also sufficient to trigger left-right habenular patterning (Lekk et al., 2019). The parapineal organ appears to regulate asymmetry of the habenulae by modulating different processes at different times (Lekk et al., 2019), but studies have focused on embryos and early larval stages. Indeed, it has been considered that the parapineal organ of teleosts is present only at embryonic/larval stages and then degenerates or “regress” during development (Friedrich-Freksa, 1932; Holmgren, 1965). However, studies in a large number of teleost species reveal that the parapineal organ in the adult brain is present (at least at the resolution of histology) in some species but absent in an equivalent number of others, although the reason for this variability is unclear (Supplementary Table S1) (Borg et al., 1983; Yañez et al., 1996; Confente et al., 2008; Herrera-Pérez et al., 2011; Birba et al., 2014; Rincón Camacho et al., 2016).

The lack of experimental approaches investigating the ontogeny of the parapineal organ using sensitive techniques beyond histology (e.g., molecular markers) has not allowed the question of the presence, absence or regression of the parapineal organ in teleosts to be adequately addressed. In fact, in zebrafish, one of the most widely used teleost models in the field of brain asymmetry, despite extensive knowledge about embryonic and larval development, practically nothing is known about the post-larval development and fate of the parapineal organ and its profuse connectivity in the adult brain. In this study, we address this question. To overcome the difficulties of observing the adult parapineal organ through classical histological techniques, we used the transgenic zebrafish line Tg(*foxd3::GFP*) which expresses the green fluorescent protein (GFP) in the pineal complex to perform a morphological and neurochemical ontogenic study of the parapineal organ from 7 days post-fertilisation to adulthood. We used 1) immunofluorescence and confocal imaging to observe and characterise parapineal cells and their projections, 2) *in vivo* focal electroporation to delineate the morphology of individual cells, and 3) transmission electron microscopy (TEM) combined with pre-embedding immunolabelling to visualise the ultrastructure of parapineal cells and their synaptic connections. We found that parapineal cells are present in the adult zebrafish brain and establish synaptic connectivity with habenular cells. However, in late juvenile stages, the parapineal is incorporated into the left habenula and loses its organ configuration, becoming a dispersed arrangement of cells. We also report that parapineal cells show ultrastructural and neurochemical features that suggest they belong to a cellular lineage distinct from other epithalamic cells. Finally, we discuss the implications of these findings in an evolutionary context.

## Methods

### Zebrafish lines and maintenance

Zebrafish (*Danio rerio*) lines used in this study were wild-type Tübingen and transgenic Tg(*foxD3::GFP*) (Gilmour et al., 2002). Embryos were obtained by natural spawning, raised at 28°C in standard embryo medium (E3) and staged according to age and morphology. Experiments were performed at 7, 9, 14, 21 and 30 days post-fertilisation (dpf) (larval and juvenile stages), and at 8 months post-fertilisation (mpf) and 1.5–2 years post-fertilisation (adult stages). All experimental procedures and animal care protocols were reviewed and approved by the Bioethics Committee on Animal Research of the Faculty of Medicine, University of Chile (CBA #0820 FMUCH).

### Pre-embedding GFP-immunolabelling

Adult transgenic zebrafish were anesthetised with tricaine (5%) and euthanised by quick decapitation. Heads were fixed in 4% paraformaldehyde (PFA)/0.05% triton in 0.1 M saline phosphate buffer pH 7.4 (PBS) for 1 h at room temperature (RT). Brains were dissected and post-fixed in 4% PFA/0.3% glutaraldehyde in PBS for 3 h at RT. Fish with strong expression of GFP in the parapineal were selected and cut into 50 µm-thick sections on a vibratome. Sections were collected, incubated in 1% sodium borohydride in PBS, dipped in cryoprotectant (25% sucrose and 3.5% glycerol in PBS), permeabilised in liquid nitrogen, and blocked with 3% normal goat serum. After blocking, sections were incubated for 48 h at 4°C with mouse anti-GFP primary antibody (Merck, 1:500), for 1 h in secondary biotinylated goat anti-mouse IgG, and then transferred to an avidin/biotin/peroxidase solution (1:50 A and 1:50 B, Vectastain Elite ABC kit) for 1 h in the dark. The tissue was rinsed and incubated in 0.022% diaminobenzidine (Merck)/0.003% hydrogen peroxide in PBS for 5 min at RT to visualise the immunostaining.

Transgenic larvae showing a strong expression of GFP in the parapineal organ at 7 dpf were selected, anaesthetised with tricaine (1%), fixed in 2% glutaraldehyde/2% sucrose, and treated with potassium cyanide, glycine, ammonium chloride and sodium borohydrate. A GFP photo-oxidation protocol with oxygen-enriched 2 mg/ml diaminobenzidine (DAB) illuminated using a 100W mercury lamp was performed as previously described (Grabenbauer et al., 2005).

### Embedding and transmission electron microscopy

GFP-immunoreactive sections were post-fixed in 1% osmium tetroxide, dehydrated and flat embedded in 100% Epon between Aclar sheets (Ted Pella) for adult sections or in a Beem® embedding capsule (Electron Microscopy Sciences) when larvae. Thin (70 nm) sections were obtained using a Leica Ultracut R ultramicrotome (Leica®) with a diamond knife, attached to mesh copper grids, and counterstained with 1% uranyl acetate. Ultrastructural analysis was performed using a Philips Tecnai 12 (Biotwin) transmission electron microscope at 80 kV. Electron micrographs were captured using an SIS CCD megaview G2 camera (Olympus©) with the iTEM Olympus Imaging Solution software.

### Immunohistochemistry

Whole-mount immunostaining was performed for animals at 7, 9, 14, 21 and 30 dpf. Fishes were euthanised by overexposure to tricaine (1%) and fixed by immersion in 4% PFA/PBS overnight. Specimens were extensively washed in PBS 1x, brains were dissected under a stereomicroscope and immunostained as previously described (Westerfield, 1995) using 10% Goat Serum, 1% DMSO, 0.5% Triton, in PBS as blocking reagent. A mix between Hoechst 33258/DAPI was used as nuclear counterstaining. Immunostaining was carried out in brain sections for zebrafish juveniles and old adults. Fishes were exposed to 5% tricaine and euthanised by quick decapitation. Heads were removed and fixed by immersion in 4% PFA/PBS for 2 h. Brains were dissected and post-fixed with the same fixative for 24 h at 4°C. Horizontal free-floating sections (50 µm) were obtained using a vibratome (Leica Biosystems, Nussloch GmbH), pre-incubated in NGS-blocking solution (see above) and then incubated with primary antibody overnight. After extensive washes with PBS, sections were incubated with the secondary antibody for 2 h at RT. Finally, sections were mounted on gelatine-coated slides using an anti-fade solution with DAPI (ThermoFisher) for nuclear counterstaining. Primary antibodies used were rabbit anti-serotonin (1:100, Merck), goat anti-substance P (1:50, Santa Cruz Biotechnology), mouse anti-zpr1 corresponding to Arrestin 3a (1:50, Zirc), mouse anti-zpr3 corresponding to Rod opsin (1:50, Zirc, Gao et al., 2022), mouse anti-tachykinin (1:50, Santa Cruz Biotechnology), mouse anti-GFP (1:500, Merck) and rabbit anti-GFP (1:500, Thermofisher). Alexa 488/647-conjugated (1:200, Thermofisher) were used as secondary antibodies.

### Focal electroporation

Focal electroporation was performed as previously described (Tawk et al., 2009) using PCS2-gap43-mCherry (1.5–2 μg/μl) and pCMV-tdTomato (Clontech®, 1.5–2 μg/μl) plasmids. Injection needles were pulled from glass borosilicate capillaries (1.2 mm OD, 0.94 mm ID, with filament, Warner Instrument), and were used to deliver DNA into one (or a few) cells through the application of five pulses of 25 V, each lasting 2 ms, using a Grass DS9 stimulator and silver electrodes. After electroporation, embryos were removed from the agarose, allowed to recover, and raised in standard medium until 7 dpf.

### Confocal image acquisition and processing

For *in vivo* imaging, embryos were anaesthetised with tricaine (0.003%) and mounted in custom-made acrylic chambers filled with 1% low melting point agarose. Samples were imaged on a Volocity ViewVox® Spinning disc (Perkin Elmer®) confocal module coupled to a Zeiss Axiovert 200 microscope, using Plan-Apochromat ×40/1.2W (pixel size 0.166 µm) or 63x/12W (pixel size 0.104 µm) objectives with lasers 488/520; 568/600 and 647/697 nm (λexc/λem). Immunostained samples were imaged on a Fluoview1000 Spectral confocal microscope (Olympus®) using Plan-Apochromat ×40 and ×60 objectives and lasers 405, 473, 559, 635 (λexc). In both cases, the z-thickness of the optical section was 0.5 µm. Fluorescence intensity image projections (Z-projections) were obtained using the ImageJ program (Schneider et al., 2012) and its extension Fiji. As the cytoplasmic Tg(*foxd3::GFP*) signal was very intense at the soma level but low at the axonal projections, which were very thin and scattered, the GFP signal intensity was intentionally increased at the acquisition stage so as not to lose the definition of the finer parapineal projections. This resulted in an unwanted saturation of the GFP signal that reduced the definition of the parapineal somas, but did not affect the ability to count the parapineal cells, as samples were always counterstained with the nuclear marker Hoechst 33258/DAPI (see below).

### Manual segmentation and three-dimensional models

**FIGURE 1:**
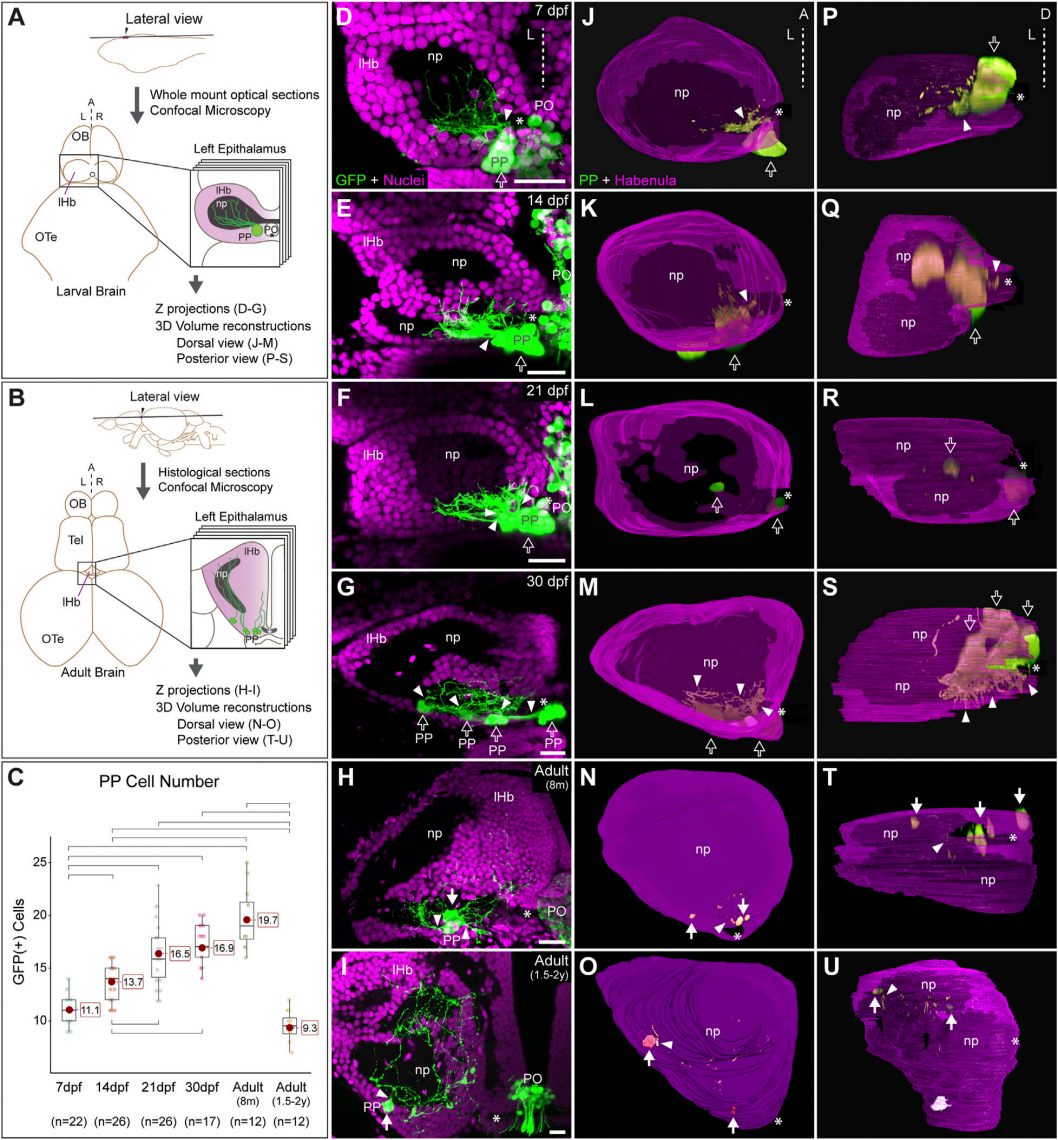
Post-hatching ontogeny of parapineal morphology and connectivity in zebrafish. **(A,B)** Schemes of larval **(A)** and adult **(B)** zebrafish brains showing the overall anatomy of the epithalamus in dorsal views and the position of optical and histological sections analysed in the rest of the panels. The left side of the epithalamus is highlighted and enlarged in a square containing the left habenula (magenta) and the parapineal (green). The position of the habenular commissure is indicated with an asterisk. **(C)** Box plot showing the quantification of GFP(+) parapineal cells in larvae (7, 14 and 21 dpf), juveniles (30 dpf) and adults (8 mpf and 1.5–2 years old). N for each group are shown below the x-axis. Significance was set to *p* < 0.001 (upper square brackets) or *p* < 0.01 (lower square brackets). The red circle on each bar is the mean for the group and its value is indicated with red square labels. The horizontal line in the box is the median of the group. **(D–I)** Ontogeny of parapineal morphology and connectivity revealed by immunofluorescence against GFP (green) and DAPI/Hoechst staining (magenta) in Tg(*foxd3::GFP*) zebrafish. Images correspond to dorsal views of representative confocal z-stacks maximum projections of the left epithalamus at 7 dpf **(D)**, 14 dpf **(E)**, 21 dpf **(F)**, 30 dpf **(G)**, 8 months **(H)** and 1.5–2 years old **(I)**, with anterior to the top, left to the left and the midline (dotted line) towards the right side of the panel. Arrowheads indicates the site where parapineal projections emerge, often forming a thick bundle at earlier stages. Empty arrows indicate the parapineal cell body as long it is recognizable as a single large cluster. White arrows indicate parapineal cell groups scattered along the posterior border of the left habenula. **(J–O)** Dorsal views of 3D reconstructions of the left habenula (magenta = cell bodies; black = neuropil) and parapineal (green) at 7 dpf **(J)**, 14 dpf **(K)**, 21 dpf **(L)**, 30 dpf **(M)**, 8 months **(N)** and 1.5–2 years old **(O)**, with anterior to the top, left to the left and the midline (dotted line) towards the right side of the panel. **(P–U)** Posterior views of 3D reconstructions of the left habenula (magenta = cell bodies; black = neuropil) and parapineal (green) at 7 dpf **(P)**, 14 dpf **(Q)**, 21 dpf **(R)**, 30 dpf **(S)**, 8 months **(T)** and 1.5–2 years old **(U)**, with dorsal to the top, left to the left and the midline (dotted line) towards the right side of the panel. Abbreviations: A (anterior), D (Dorsal), L (left), lHb (left habenula), np (habenular neuropil), OB (Olfatory Bulb), OTe (Optic Tectum), PO (pineal organ at the level of the stalk), PP (parapineal), R (right), Tel (Telencephalon). Scale bars, 10 µm.

Three-dimensional models shown in Figure 1 were built from confocal GFP/nuclei images captured as explained in the previous section. Manual habenular and neuropil contour segmentation was performed by outlining the object contour in each z-slide as a closed polygon using a digital Pen CTE-440 tablet (Wacon®). Next, stacks of 2D binary ROIs were generated with a custom-made macro written for the Image SXM software program (Barrett, 2014) for both the habenula and the neuropil. Last, binary ROIs were used with the original GFP channel to generate a 3D surface mesh and voxel-intensity models using the SCIAN-Soft, a custom-built software platform programmed in IDL 7.1.2 (ITT/Harris).

### Quantification of parapineal cell number and statistics

Individual GFP-positive parapineal cells were identified with Hoechst 33258/DAPI counterstaining and manually counted in confocal stacks obtained from immunofluorescence experiments on Tg(*foxd3::GFP*), using the Cell Counter plugin for ImageJ/Fiji (see above). Statistical analyses comprised first a Shapiro-Wilk to test the normality of the data, a non-parametric test Kruskal-Wallis, and a post hoc Bonferroni Mann-Whitney. Significance was set to either *p* < 0.001 or *p* < 0.01. Boxplot of GFP(+) parapineal cells during ontogeny was build through ggstatsplot package (Patil, 2021). All statistical analysis were performed using R Statistical Software (version 4.2.1) and R studio (version 2022.02.3).

## Results

### The zebrafish parapineal incorporates into the left habenula and loses its organ configuration during ontogeny

We performed an ontogenic series of anti-GFP immunofluorescence combined with DAPI/Hoechst fluorescent nuclear staining in Tg(*foxd3::GFP*) fishes. We found GFP-positive cells belonging to the parapineal organ in all examined stages, but their topological organisation changed markedly over time. From 7 to 21 dpf, parapineal cells and their projections mostly retained the topological organisation already described in the larvae (Concha et al., 2003; Gamse et al., 2003). Parapineal cell bodies arranged as a compact cluster at the medial and posterior border of the left dorsal habenula in proximity to the habenular commissure (empty arrows in Figures 1D–F,J–L,P–R). Projections emerged together from the parapineal body forming a compact bundle that crossed the habenular commissure in the posterior-to-anterior direction (arrowheads in Figures 1D–F; see also Signore et al., 2009), to branch profusely into the dorsal-most habenular neuropil, primarily in its posterior domain (Figures 1D–F). At 21 dpf, the configuration of a single-bundle of parapineal projections began to change, with the presence of a few neighbouring fascicles emerging from a still tightly clustered parapineal body (arrowheads in Figure 1F). From 30 dpf (juvenile stage) onwards, parapineal cells detached from each another, either as individuals or in small groups located along the posterior border of the left habenula, adjacent to the neuropil (white arrows in Figures 1G,M,S). However, at 30 dpf, it was still possible to recognise the original parapineal body as the largest agglomerate of cells in a medial and posterior location (Figures 1G,M,S). Parapineal projections reached the same domain of the left dorsal habenular neuropil, but due to cell separation, they entered the habenula as scattered fascicles crossing the habenular commissure (arrowheads in Figures 1G,M). In the adult brain, at 8 months, it was no longer possible to recognise a main group of medially positioned parapineal cells and instead individual cells, or small groups, were observed more laterally but still close to the habenular commissure and the neuropil domain adjacent to it (white arrows in Figures 1H,N,T). Parapineal projections, after crossing and moving away from the commissure, blended into the dorsal neuropil and between habenular cells. Later, at 1.5–2 years post fertilisation the parapineal cell bodies were dispersed and closely surrounded by habenular cells, located mainly along the posterior border of the neuropil and no longer found near the habenular commissure (Figures 1I,O,U). Parapineal projections were large and profusely branched, extending over a wide area of the dorsal-most neuropil of the left dorsal habenula. Although we found variability among individuals regarding whether the parapineal cells were found singly or as small groups of two or three within the left habenula, the overall dispersed morphological pattern was conserved. Quantification of GFP-positive parapineal cells (see *Material and methods* and Figure 1C) revealed an increase in cell number during the larval stages between 7 and 21 dpf, after which the number of GFP-positive cells remained stable with no statistically significant changes through juvenile (30 dpf) to the 8 mpf adult brain. Later, in the 1.5–2 years old adults, a decrease of cells was observed to reach levels comparable to those observed in the early larva (7 dpf) (Figure 1C). In summary, parapineal cells are present throughout ontogeny. However, their topological arrangement changes markedly over time, losing their clustered organisation in the adult brain.

### Parapineal cells are unipolar neurons forming synapses with the left habenular neuropil

**FIGURE 2:**
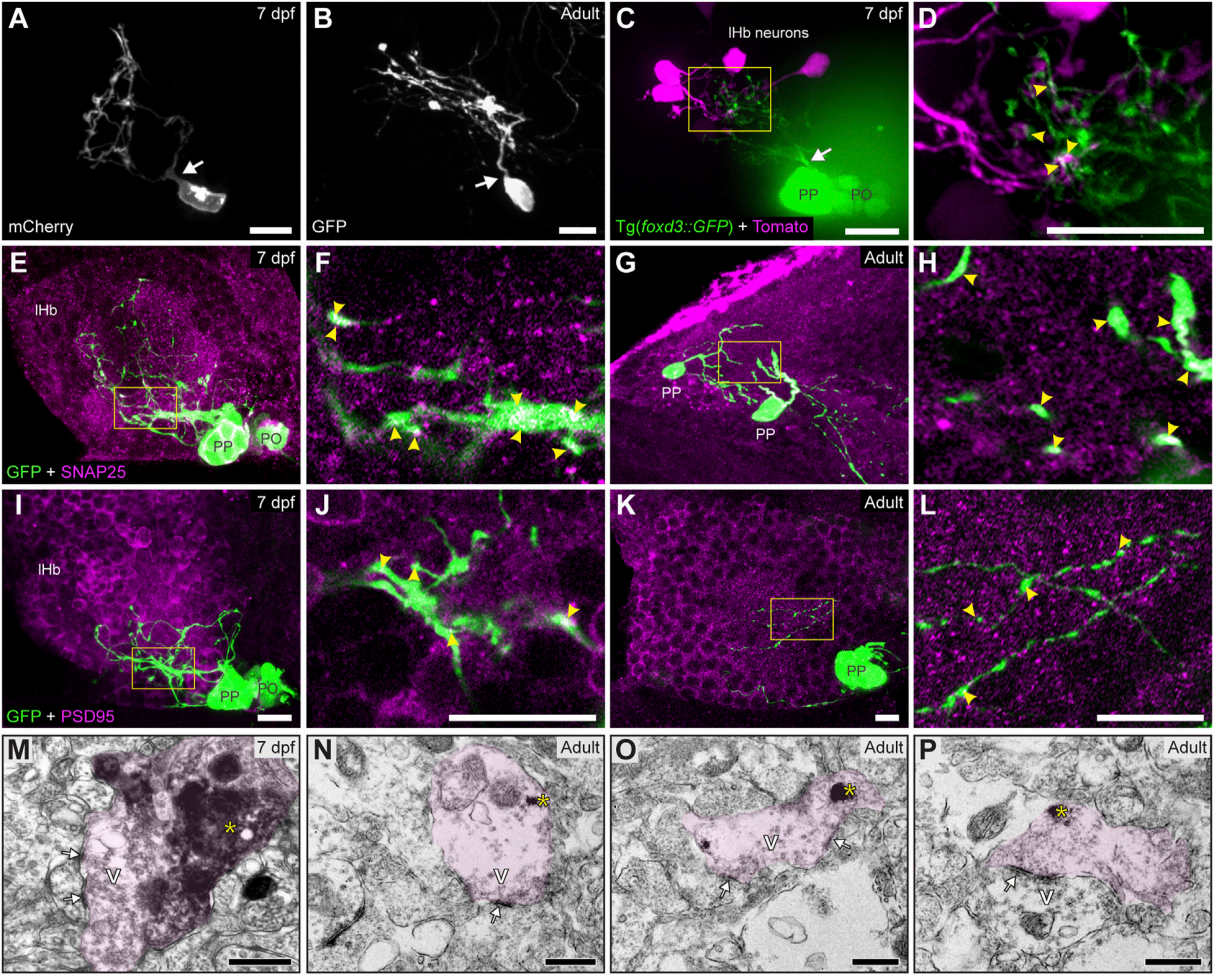
Contacts and synapses between parapineal cells and the left habenular neuropil in the larva and adult zebrafish. **(A,B)** Dorsal views of confocal z-stack maximum projections showing single parapineal cells expressing mCherry in a living larva at 7 dpf **(A)** and GFP in a section of the 1.5–2 years old adult brain **(B)** after immunostaining against this fluorescent protein. At both stages, parapineal cells show a characteristic pear-shape cell body with a single projection emerging from it (white arrows) and then branching profusely. **(C,D)** Dorsal views of confocal z-stack maximum projections showing 4 habenular neurons expressing the fluorescent protein Tomato (magenta) in a living 7 dpf Tg(*foxd3::GFP*) larva expressing GFP in the pineal complex (green). Projections emerge from the parapineal body as a bundle (white arrow), then branch in the left habenula to intermingle with the dendritic arbour of the labelled habenular neurons. The image in **(D)** corresponds to a z-plane of the yellow rectangle depicted in **(C)**, showing the punctuated zones where the fluorescent signals corresponding to parapineal projections (green) closely associate with the fluorescence signals corresponding to habenular dendrites (magenta). **(E–L)** Dorsal views of immunofluorescence against presynaptic (SNAP25) and postsynaptic (PSD95) markers in the epithalamus of larval (7 dpf) and adult (1.5–2 years old) zebrafish. Images correspond either confocal z-stack maximum projections **(E,G,I,K)** or to single z-planes (**F,H,J,L**; corresponding to the yellow rectangles depicted in **E,G,I,K**, respectively) of larval **(E,F,I,J)** and adult **(G,H,K,L)** animals. Yellow arrowheads point to zones where the fluorescent signal from to parapineal projections (green) closely associate with the immunofluorescence signal of the synaptic protein (magenta). **(M–P)** TEM images showing the synaptic relation between parapinal cells and the habenular neuropil at larval (7 dpf) **(M)** and adult (1.5–2 years old) **(N–P)** stages. GFP-immunopositive terminals of parapineal projections showing black DAB precipitates (yellow asterisks) are shaded in pink. These terminals show synaptic vesicles (v) and synaptic densities (white arrows) both in larvae and adults. Abbreviations: lHb (left habenula), PO (pineal organ at the level of the stalk), PP (parapineal). Scales bars, 10 µm **(A–L)**, 500 nm **(M–P)**.

Despite the profuse parapineal projection toward the left habenula, synaptic communications between them have not been described in detail. To study this, we used three complementary techniques. First, we performed focal electroporation to differentially label individual parapineal cells and small groups of habenular cells and examine *in vivo* their morphology and contact. In both larvae (Figure 2A) and adults (Figure 2B), parapineal cells exhibited a stereotypical unipolar morphology with a pear-shaped cell body and a single process extending towards and branching into the left habenular neuropil, intermingling with the habenular dendritic extensions. The close spatial association between the fluorescent signal of parapineal projections and habenular processes (Figures 2C,D; arrowheads in Figure 2D) suggested a functional contact between them. Thus, we performed immunofluorescence against pre- and post-synaptic proteins to investigate whether these contacts could correspond to synapses. The presynaptic protein SNAP25 was found profusely in larvae (Figures 2E,F; arrowheads in Figure 2F) and adults (Figures 2G,H; arrowheads in Figure 2H), both in the habenular neuropil and parapineal cells, where it was detected in projections and very strongly in the cell body membrane (Figures 2E,G). The postsynaptic marker PSD95 was also found in parapineal cells. Like SNAP25, PSD95 was detected in both projections and cell bodies, but the signal was weaker (Figures 2I–L). Finally, to directly demonstrate the presence of mature synapses, we analysed the ultrastructure of parapineal neurons and their projections by TEM in the 7 dpf larva and the adult brain. Synapses were identified by the characteristic dark and thickened appearance of the postsynaptic density (arrows in Figures 2M–P) together with the presence of vesicles in the presynaptic active zone (marked as “V” in Figures 2M–P). Parapineal projections of transgenic larva and adult fishes were identified through the pre-embedding immunolabelling of GFP (pink shadows in Figures 2M–P; see *Material and methods*) allowing the observation of synaptic contacts between presynaptic GFP-positive parapineal projections and postsynaptic GFP-negative fibres, very likely belonging to habenular cells (Figures 2M–O). In adults, we also observed a few synaptic contacts where GFP-positive fibres acted as postsynaptic (Figure 2P), suggesting that parapineal neurons could establish bidirectional communication with the left habenular neuropil. Altogether, these findings demonstrate that in zebrafish, parapineal cells form morphologically mature synapses with the left habenular neuropil from larval stages (at least as early as 7 dpf) to adulthood.

### Parapineal cells are immunoreactive to neurotransmitters and neuropeptides during ontogeny

**FIGURE 3:**
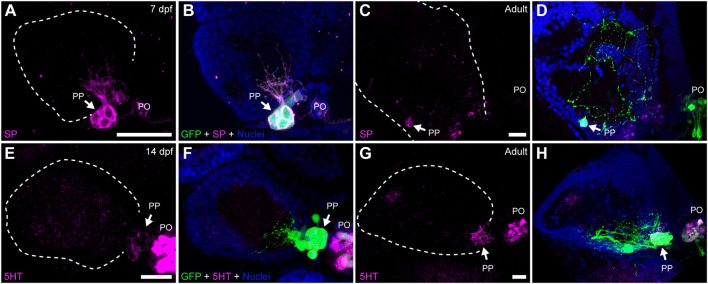
Parapineal cells show immunoreactivity against substance P and serotonin during ontogeny. Indirect immunofluorescence against substance P (SP) **(A–D)** and serotonin (5HT) **(E–H)** in larval (7 and 14 dpf) and adult (1.5–2 year old) Tg(*foxd3::GFP*) zebrafish. Images correspond to dorsal views of confocal z-stack maximum projections, with anterior to the top and left to the left, showing the fluorescence signal corresponding to SP (magenta in **(A,C)**) and 5HT (magenta in **(E,G)**), or the merge fluorescence signals that also include the GFP of the pineal complex (green) and the DAPI/Hoechst nuclear staining providing the left habenula tissue context (blue) **(B,D,F,H)**. Arrows indicate parapineal neurons immunoreactive to SP and 5HT. Abbreviations: PO (pineal organ at the level of the stalk), PP (parapineal). Samples: 7 dpf SP (*n* = 5), adult SP (*n* = 5), 14dpf 5HT (*n* = 5), adult 5HT (*n* = 5). Scale bars, 10 µm.

Previous studies revealed that cells within structures homologous to the teleost parapineal in lampreys (parapineal organ and ganglion) and lizards (parietal eye) show immunoreactivity against neurochemicals such as serotonin (5HT), substance P (SP), neuropeptide Y (NPY), galanin, choline acetyltransferase (ChAT) and Υ-amino butyric acid (GABA) (Engbretson, 1992; Yáñez et al., 1999). We thus investigated whether zebrafish parapineal cells also express some of these neurochemicals by performing immunofluorescence in larva and adult transgenic Tg(*foxd3::GFP*) (Figure 3; Supplementary Table S2). We found SP immunoreactivity in parapineal cells and their projections at 7 dpf (Figures 3A,B) and in adults (Figures 3C,D). Supporting this finding, we observed immunoreactivity against the SP-precursor tachykinin (TAC) in parapineal cells (Supplementary Figure S1). Noteworthy, SP appeared to be present in all the GFP-positive parapineal cells, but TAC was not. Among the classic neurotransmitters, we found 5HT immunoreactivity in a subset of parapineal cells from 14 dpf (Figures 3E,F) to adulthood (Figures 3G,H). Interestingly, immunoreactivity against 5HT changed depending on the time of the day in which the fish were processed. The strongest 5HT signal was found in samples collected late in the afternoon, while samples obtained during the morning exhibited the weakest signal (data not shown). Both 5HT and SP were also observed in pineal cells (Figures 3E–H). Finally, we could not detect immunoreactivity for NPY, GABA, ChAT nor TH in the zebrafish parapineal (Supplementary Table S2).

### Parapineal cells show ultrastructural and immunohistochemical features suggestive of a distinct cell lineage

**FIGURE 4:**
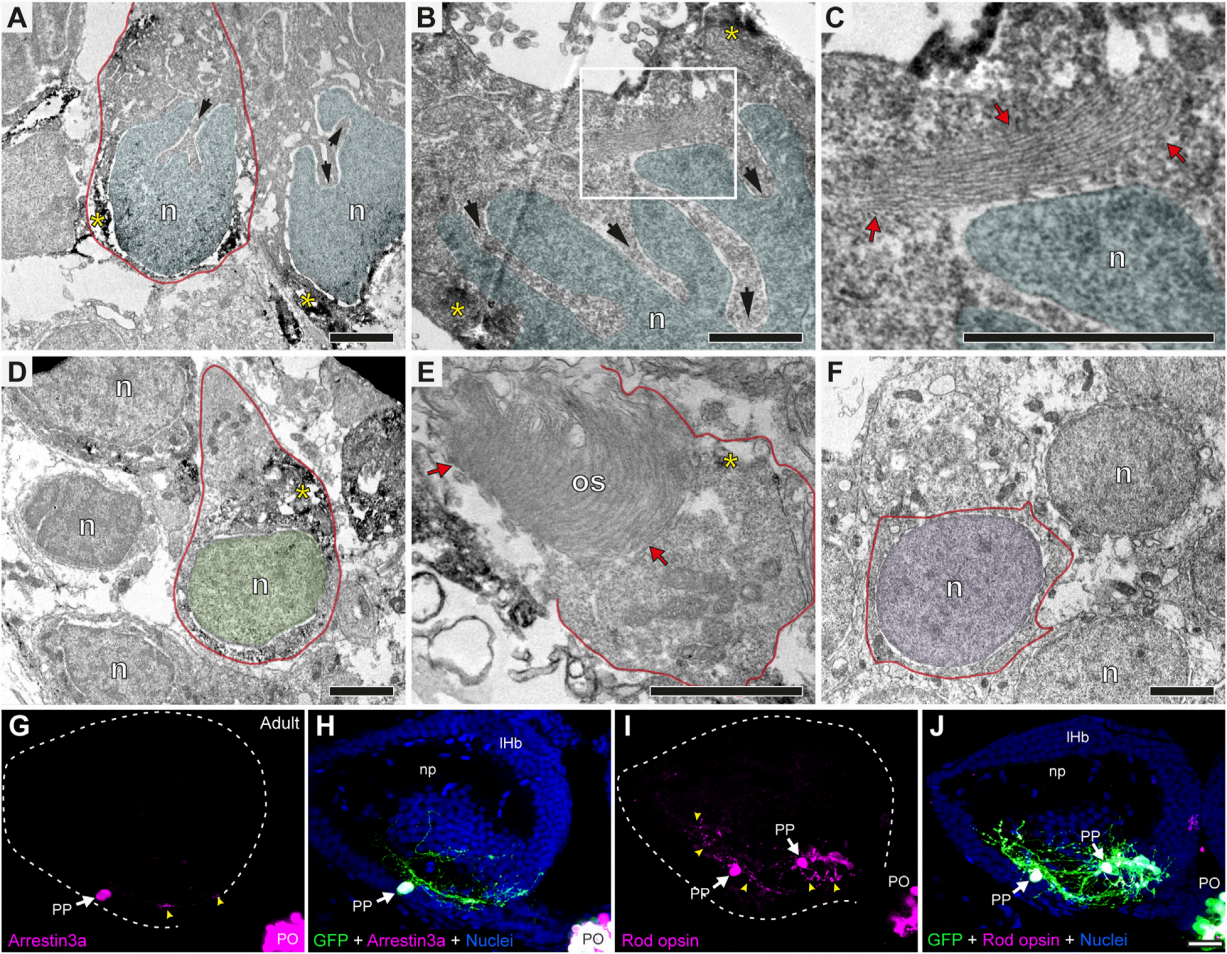
Parapineal cells show ultrastructural and immunohistochemical features distinctive from other epithalamic cells and suggestive of a photosensitive function in the adult zebrafish. **(A–F)** TEM images showing the cell types found in the adult (1.5–2 years old) zebrafish epithalamus: parapinealocytes (**(A–C)**; nucleus labelled in light blue), pinealocytes (**(D,E)**; nucleus labelled in green) and habenular cells (**(F)**; nucleus labelled in pink). GFP-immunopositive parapinealocytes and pinealocytres display black DAB precipitates (yellow asterisks). The cell membrane is depicted as a red line. Parapinealocytes show a pear-shape cell body (red outline in **(A)**), a nucleus with indentations or clefts (black arrows in **(A,B)**), and a lamellar structure near the nucleus (red arrows in **(C)**, which correspond to a high magnification view of the white rectangle depicted in **(B)**). Pinealocytes also show a pear-shape cell body (red outline in **(D)**) but the nucleus lacks indentations and shows an external segment characteristic of photoreceptors (red arrows in **(E)**). The cell body of habenular neurons is mostly occupied by its round and regular nucleus **(E)**. **(G–J)** Immunofluorescence against Arrestin3a **(G,H)** and Rod opsin **(I,J)** in adult (1.5–2 years old) Tg(*foxd3::GFP*) zebrafish. Images correspond to dorsal views of confocal z-stack maximum projections, with anterior to the top, showing in the left epithalamus the fluorescence signal corresponding to Arrestin3a (magenta in **(G)**) and Rod opsin (magenta in **(I)**), or the merge fluorescence signals that also include the GFP of the pineal complex (green) and the DAPI/Hoechst nuclear staining that provides the left habenula tissue context (blue) **(H,J)**. White arrows and yellow arrowheads indicate immunoreactive parapineal cell bodies and projections, respectively. Abbreviations: n (nucleus), os (outer segment). PO (pineal organ at the level of the stalk), PP (parapineal). Adult samples: Arrestin3a (*n* = 4), Rod opsin (*n* = 6). Scale bars, 1 µm **(B, C)**, 2 µm **(A,D–F)** and 10 µm **(G–J)**.

To understand to what extent the parapineal, pineal and habenular cells are morphologically distinguishable, we performed TEM. We found that, while the pear-shaped cell body with the nucleus at the widest part resembled a GFP-positive photoreceptive pinealocyte (Figure 4A), parapineal cells showed conspicuous nuclear indentations or clefts, often giving rise to a “C-shaped” nucleus (arrows in Figures 4A,B). No indentations were found in pinealocytes (Figure 4D; see also Laurà et al., 2012) or in habenular cells, whose nuclei were remarkably rounded with little surrounding cytoplasm (Figure 4F). In addition, unlike pinealocytes, parapineal cells did not have large or dense granules (Figure 4D; see also Laurà et al., 2012). Furthermore, we could not detect typical ultrastructural features of photoreceptors in parapineal cells, such as outer and inner segments with 9 + 2 cilia and mitochondria, which are usually observed in pinealocytes (red arrows in Figure 4E). Nevertheless, in the cytoplasm of parapineal cells, we observed elongated lamellar structures with a small number of piled membranes (red arrows in Figure 4C). To study whether this membranous arrangement might relate to a photoreceptive structure, we performed immunofluorescence against two photoreceptor markers Arrestin3a (ZPR1, double green/red cones marker) and Rod opsin (ZPR-3, outer segments of green-cones marker, Gao et al., 2022). Consistent with this idea, both photoreceptor markers were detected in parapineal cell bodies at the adult stage (Figures 4G–J). Taken together, these findings indicate that the parapineal cell lineage shows ultrastructural and immunohistochemical features distinct from those of other epithalamic cells. They also indicate a possible parapineal photosensitive function.

## Discussion

### The adult zebrafish lacks a parapineal organ but has parapineal cells

This study is the first detailed ontogenic analysis of the parapineal organ in a teleost, from larval stages to adulthood. Our findings in zebrafish reveal that up to 21 dpf the parapineal is recognisable as an organ, whereas thereafter it is no longer a distinguishable morphological unit as its cells lose contact with each other and integrate into the left dorsal habenula, either as single cells or in small groups. Thus, the parapineal as an “organ” is present in embryos, larvae and juveniles but is absent in adult zebrafish. This absence is not due to true ontogenetic regression mediated, for example, by apoptosis. In fact, parapineal cells survive throughout the life (larvae, juveniles and adults) of the zebrafish and maintain connections with the left habenular neuropil, only showing a reduction in number in the old adult brain possibly due to the aging process. A similar integration mechanism has recently been described in another teleost, the Japanese medaka *Oryzias latipes* whose parapineal is also incorporated into the left habenula, but with a different topology and timing (Ishikawa et al., 2015). The parapineal cells of medaka are integrated into the left caudomedial habenular subnucleus as a compact cluster (termed the parapineal domain of the habenula) that is maintained at least until old juvenile fish. Moreover, in medaka, the incorporation of the parapineal organ is complete as early as Iwamatsu Stage 30 (Ishikawa et al., 2015), which corresponds to 33 hpf in zebrafish, according to our time normalisation method (Signore et al., 2009). This timing is strikingly earlier than the 21–30 dpf, when the same process starts in zebrafish. Remarkably, a common feature between the two modes of parapineal incorporation is the persistence of the connectivity between parapineal cells and the left habenula. Nevertheless, given that the later stage analysed in medaka corresponds to an old juvenile (Ishikawa et al., 2015), it remains to be determined whether any configuration changes occur later in this species.

### Cell displacement and intermingling as mechanisms of morphological variation in the evo-devo of parapineal-habenular interactions

The close relationship between the parapineal organ and the left habenula is a conserved feature in vertebrates beyond teleosts (Guglielmotti and Cristino, 2006). In light of this study, it is of particular interest that in lampreys, the parapineal organ is related to an accessory ganglion (termed the parapineal ganglion) which is a part of the left habenular nucleus that migrates rostrally during development (Studnička, 1893; Meiniel and Collin, 1971). Meiniel et al. (1971) advanced an evolutionary hypothesis to explain why the parapineal organ is very rich in ganglion-like cells and almost devoid of photoreceptors in *Salmo gairdneri* (and probably in other teleosts), whereas the opposite is true in the lamprey *Lampetra planeri*. They proposed the incorporation of ganglionic elements belonging to the central nervous system into the parapineal organ. The co-existence of the parapineal ganglion with an almost exclusively photoreceptive parapineal of *Lampetra* would be the preliminary step in this integration process (Meiniel and Collin, 1971). Furthermore, in a study in the teleost *Phoxinus phoxinus*, (Ekstrom et al., 1987) hypothesised that migrating pineal complex cells invading the habenular nuclei form a “displaced” parapineal organ within the habenula. They also proposed that this mechanism could explain the absence of the parapineal organ in some teleosts and the presence of “displaced” pinealocytes immunoreactive to S-antigen and serotonin in the medial habenular nucleus of rodents (Steinbusch, 1984; Korf et al., 1986). Consistent with this idea, the left habenula of Atlantic salmon (*Salmo salar*) shows a small distinct dorsal cellular domain expressing the vertebrate ancient opsin and melanopsin, which is also light reactive (Eilertsen et al., 2021). Taken together, these observations strongly suggest that during ontogeny, the habenula and parapineal organ are not only closely interconnected through signalling and patterning as revealed by developmental studies of epithalamic left-right asymmetry in zebrafish (Gamse et al., 2003; Concha et al., 2009; Lekk et al., 2019), but also through cell displacement and intermingling. Since these processes are highly dependent on time, topology, geometry, genetics and developmental peculiarities of each animal, the large spectrum of evolutionary variations observed even in closely related species becomes very likely and expected.

### Parapineal cells form a distinct lineage from other epithalamic cells

Developmental studies have shown that the fate of parapineal cells is established at embryonic stages in the most anterior cellular domain of the pineal complex (Snelson et al., 2008a; 2008b; Clanton et al., 2013; Khuansuwan et al., 2016). Shortly thereafter these cells undergo morphogenetic transformations that physically separate them from the pineal anlage and position them on the left side of the epithalamus (Concha et al., 2003; Snelson et al., 2008b; Roussigné et al., 2018) where they show a pattern of connectivity that differ from that of pineal and habenular cells (Concha et al., 2000, 2003; Concha and Wilson, 2001; Gamse et al., 2003; Turner et al., 2016). Our morphologic, ultrastructural, and neurochemical analyses of larval and adult zebrafish provide further evidence that, despite a common origin from the pineal complex, the parapineal is a distinct cell lineage from the pineal, and that despite its incorporation into the left habenula it is also distinct from the habenular lineage. The parapineal cells show indentations in their nuclei that are not present in pineal and habenular cells, and small lamellar structures suggesting a possible photosensitive function that is supported by parapineal immunoreactivity against the photoreceptor markers Arrestin 3a and Rod opsin, but clearly distinct from the outer segments observed in pinealocytes. These results support the proposal of Ekström and Meissl (2003) for the evolution of pineal complex cells through developmental changes in fate restriction, rather than the classic paradigm of ontogenetic regression of photoreceptive parts in the pineal cell. In this context, the presence of nuclear clefts is remarkable and may be related to the ontogenic history of parapineal cells. Nuclear physical properties such as morphology and deformability, have been associated with cellular functions like gene expression, genome integrity and cell behaviour, as forces acting on the cytoskeleton and nucleoskeleton can regulate chromatin remodelling (Makhija et al., 2016). Indeed, nuclear shape is the reflection of cell shape and its mechanical environment, and it has even been proposed that it may reflect the history of cumulative shape changes experienced by the cell in its ontogeny (Lele et al., 2018). Probably, the most important phenomenon impacting cell/nuclear shape is cellular movement, and since cell migration is a hallmark of the parapineal lineage that is not observed in pineal and habenular cells, it is tempting to speculate that the parapineal “deformed” nucleus is, somehow, the ontogenetic mark of the convoluted travel history of these cells.

### Parapineal cells form part of a left habenular circuit throughout life

Zebrafish parapineal cells are unipolar neurons, with a pear shape cell body and a single neurite that enters the left dorsal habenula and branches into the dorsal-most domain of habenular neuropil. Morphologically mature synapses form between parapineal cells and habenular dendrites with pre- and post-synaptic terminals in both larvae and adults. In addition, there are few postsynaptic terminals in adults, but it should be noted that, due to the experimental protocol, our analysis was biased towards areas with an intense GPF signal, i.e., near the cell bodies. Taken together, our results strongly suggest the existence of a direct neural circuit between the parapineal and the neuropil of the left habenula, which may contain habenular dendrites and forebrain-derived afferents to the habenula. As parapineal cells show ultrastructural and neurochemical features suggestive of photosensation, it is possible to speculate that parapineal neurons, through synaptic contacts with the left habenular neuropil, may function as asymmetric local modulators of habenula-associated neural circuitry in response to light, or circadian variations in light. Indeed, 5HT, a well-described melatonin precursor in pinealocytes (Gothilf et al., 1999; Falcón et al., 2010) whose expression is circadian regulated in fish, is also observed in parapineal cells and shows apparent circadian variation in zebrafish. The observation of SP immunoreactivity in parapineal cells is also relevant for a possible parapineal modulatory activity. This decapeptide, canonically associated with pain perception in the peripheral and central nervous system and acting as a neuromodulator or neurotransmitter, has been linked to anxiety modulation, which is particularly interesting considering that the habenula is involved in the control of anxiety and fear responses in zebrafish and other animals (Okamoto et al., 2012; Mathuru and Jesuthasan, 2013; Yamaguchi et al., 2013; Jacinto et al., 2017). SP has been described in the habenular-interpeduncular circuit in many vertebrates (lampreys, teleost, amphibians, lizards and rodents) and in lizards, lampreys and the teleost *Oncorhynchus mykiss*, it is present in the parietal eye, parapineal ganglion and parapineal organ (Kemali and Guglielmotti, 1984; Ekström and Korf, 1986; Engbretson, 1992; Rodriguez-Moldes et al., 1993; Yáñez and Anadón, 1996). In addition to SP and 5HT, we could not detect the presence of NPY, GABA, ChAT or TH, unlike what is observed in the parapineal of other vertebrates, so our understanding of the neurochemical nature of parapineal-habenular communication is still very limited. Further neurochemical and neurophysiological studies are needed to elucidate this point, and to corroborate the possible modulatory activity of the parapineal on the habenula-related circuitry, which in teleosts and other vertebrates has the interpeduncular nucleus and raphe nuclei as the main efferent targets (Bianco and Wilson, 2009). These studies combined with parapineal ablation at different ontogenic stages, will help to reveal the possible role of the parapineal in animal behaviour. So far, pre-hatching parapineal ablation experiments in zebrafish result in reduced exploratory behaviour in adults (Decarvalho et al., 2013), but it is unclear whether the change is due to early disruption of habenular development or to a direct late function of the parapineal.

### Final reflections

The idea that the parapineal is absent in the brain of adult teleosts due to degeneration during development and evolution has long persisted in the field, so that this organ has often been described as a rudiment or a vestige when found in adult fish (Holmgren, 1959; Kappers, 1965; Korf, 1974; Cole and Youson, 1982). Both concepts (rudimentary and vestigial) imply, more or less explicitly, the detriment of the organ’s function, as this loss would relieve selective pressure, allowing the evolutionary regression of the structure due to its uselessness or inutility (Darwin et al., 1876; Pagel, 2002). Even accepting the word “rudimentary” to refer to any reduced organ in a broad sense (Schaffner, 1906), this terminology is hardly usable in a rigorous way and contributes to maintaining implicit assumptions unsupported by experimental evidence. The function of the parapineal organ in adult teleosts is unknown and until we know it, we cannot make any assumptions about a possible functional detriment of this organ during ontogeny or phylogeny. The comparison with the pineal organ does not help, as pineal and parapineal are different cell lineages. The contrast with homologous structures in other species such as the parietal eye of lizards, which shows an eye-like structural organisation, does not help to answer this question either, as the complexity of the parietal eye probably represents a specialisation of this animal. The only answer comes from detailed ontogenic analysis. Our study shows that parapineal cells remain throughout ontogeny although they change their organisation by incorporating into the left habenula, showing features suggestive of photosensation and retaining connectivity with the left habenula. Taken together, these results suggest a function in the adult animal. Elucidating this function is a challenge that will need to be resolved by future studies that take advantage of newly available *in vivo* functional and behavioural methodologies. Furthermore, given that parapineal cell organisation may be variable and highly changeable during ontogeny, comprehensives studies of parapineal ontogeny in other teleosts and vertebrates are needed to unravel the complexity of epithalamic development and evolution. Finally, we cannot overemphasise the importance of the temporal dimension in morphogenesis, both during ontogeny and phylogeny, as changes in the relationship between interacting cell populations during development give rise to variability and evolution of form in living beings.

## Data Availability

The original contributions presented in the study are included in the article/Supplementary Material, further inquiries can be directed to the corresponding author.
