# Supplementary material for: Ontogenesis of the asymmetric parapineal organ in the zebrafish epithalamus
Source: Front Cell Dev Biol. 2022 Dec 9;10:999265. doi: 10.3389/fcell.2022.999265 (PMC9780773; doi:10.3389/fcell.2022.999265)
Supplement: Supplementary file 1 [file DataSheet1.pdf]

Ontogenesis of the asymmetric parapineal organ in the zebrafish epithalamus

Karina Palma,[1,2┼] Iskra A. Signore,[1,2┼] Margarita M. Meynard,[1,2,3] Jazmin Ibarra,[1,2] Lorena Armijo-Weingart,[4] Marcos Cayuleo,[1,2] Steffen Härtel,[1,2,5] and Miguel L. Concha[1,2,3 *]

Supplementary Table 1: Presence/absence of parapineal organ at juvenile/adult stages in teleosts.

Teleost species / order	Presence of Parapineal Organ			Reference
	Adult	Juvenile	Unknown age	
Gnathonemus sp / Osteoglossiformes	Yes			Borg, Ekstrom & Van Veen 1983.
Anguilla anguilla / Anguilliformes	Yes			Rudeberg 1971.
Esox lucius / Esociformes	Yes			Rudeberg 1969.
Bathylagus wesethi / Osmeriformes	No			McNulty 1976.
Oncorhynchus mykiss / Salmoniformes	Yes			Yañez 1996.
Salmo gairdneri / Salmoniformes	Yes			Rudeberg 1968.
Salmo salar / Salmoniformes	No	Yes		Holmgren 1965.
Salvelinus fontanalis / Salmoniformes	No	Yes		Hill 1894; Holmgren 1965.
Salvelinus alpinus / Salmoniformes	No	Yes		Vigh-Teichmann 1991; Musæus 2009.
Catla catla / Cypriniformes	No			Dey 2003.
Labeo bicolor / Cypriniformes	Yes			Borg, Ekstrom & Van Veen 1983.
Carassius auratus / Cypriniformes			Yes	Borg, Ekstrom & Van Veen 1983.
Cyprinus carpio / Cypriniformes			Yes	Borg, Ekstrom & Van Veen 1983.
Hypophthalamichthys molitrix / Cypriniformes			Yes	Borg, Ekstrom & Van Veen 1983.
Labeo umbratus / Cypriniformes	Yes			Borg, Ekstrom & Van Veen 1983.
Phoxinus phoxinus / Cypriniformes	Yes			Vigh-Teichmann 1982.
Alburnus tarichi / Cypriniformes	No			Orgi & Oguz 2022.
Gyrinocheilus aymoneri / Cypriniformes	Yes			Borg, Ekstrom & Van Veen 1983.
Nannostomus sp / Cypriniformes	Yes			Borg, Ekstrom & Van Veen 1983.
Acanthophthalmus sp / Cypriniformes		Yes		Borg, Ekstrom & Van Veen 1983.
Carnegiella sp / Characiformes	No			Borg, Ekstrom & Van Veen 1983.
Gasteropelecus sp / Characiformes	No			Borg, Ekstrom & Van Veen 1983.
Aphyocharax anisitsi / Characiformes	Yes			Rincón-Camacho 2016.
Paracheirodon axelrody / Characiformes	Yes			Rincón-Camacho 2016.
Gymnocorymbus ternetzi / Characiformes	Yes			Borg, Ekstrom & Van Veen 1983.
Corydora sp / Siluriformes	Yes			Borg, Ekstrom & Van Veen 1983.
Heteropneustes fossilis / Siluriformes			Uncertain	Borg, Ekstrom & Van Veen 1983.
Ictalurus nebulosus / Siluriformes			Yes	Borg, Ekstrom & Van Veen 1983.
Ceratoscopelus townsendi / Myctophimorfes	No			McNulty & Nafpaktitis 1977.
Diaphus theta / Myctophimorfes	No			McNulty & Nafpaktitis 1977.
Lampanyctus ritteri / Myctophimorfes	No			McNulty & Nafpaktitis 1977.

Parvilux ingens / Myctophimorfes	No			McNulty & Nafpaktitis 1977.
Stenobrachius leucopsaurus / Myctophimorfes	No			McNulty & Nafpaktitis 1977.
Symbolophorus californiensis / Myctophimorfes	No			McNulty & Nafpaktitis 1977.
Tarletonbeania crenularis / Myctophimorfes	No			McNulty & Nafpaktitis 1977.
Triphotorus mexicanus / Myctophimorfes	No			McNulty 1976.
Gadus morhua / Gadiformes	Yes			Borg, Ekstrom & Van Veen 1983.
Zoarces viviparous / Gadiformes	Yes			Borg, Ekstrom & Van Veen 1983.
Nezumia liolepis / Gadiformes	No			McNulty 1976.
Xenentodon cancila / Beloniformes	No			Shrivastava 1972.
Oryzias latipes / Beloniformes	No	Yes		Ishikawa 2015.
Poecilia latipinna / Cyprinodontiformes	No	No		Borg, Ekstrom & Van Veen 1983.
Poecilia reticulate / Cyprinodontiformes	No	No		Borg, Ekstrom & Van Veen 1983.
Xiphophorus helleri / Cyprinodontiformes	No	No		Borg, Ekstrom & Van Veen 1983.
Epiplatys sp / Cyprinodontiformes	No	No		Borg, Ekstrom & Van Veen 1983.
Nothobranchius rachovi / Cyprinodontiformes	No	No		Borg, Ekstrom & Van Veen 1983.
Spinachia spinachia / Gasteroiformes		Yes		Borg, Ekstrom & Van Veen 1983.
Gasterosteus aculeatus / Gasteroiformes	Yes			Van veen 1980.
Nerophys ophidion / Gasteroiformes	Yes			Borg, Ekstrom & Van Veen 1983.
Helostoma temmincki / Perciformes	Yes			Borg, Ekstrom & Van Veen 1983.
Dicentrarchus labrax / Perciformes	Yes			Herrera-Perez 2011.
Trachurus japonicas / Perciformes			Yes	Ueck 1979.
Chanda ranga / Perciformes	Yes			Borg, Ekstrom & Van Veen 1983.
Cichlasoma cyanoguttatum / Perciformes	Yes			Borg, Ekstrom & Van Veen 1983.
Cichlasoma dimerus / Perciformes	Yes			Birba et al 2014.
Pelmatochromis kribensis / Perciformes	Yes			Borg, Ekstrom & Van Veen 1983.
Monodactylus argentus / Perciformes		Yes		Borg, Ekstrom & Van Veen 1983.
Perca fluvialis / Perciformes	Yes			Borg, Ekstrom & Van Veen 1983.
Stizostedion lucioperca / Perciformes			Yes	Vigh-Teichmann 1990.
Badis badis / Perciformes	Yes			Borg, Ekstrom & Van Veen 1983.
Typhlogobius californiensis / Perciformes	No			McNulty 1978.
Helicolenus hilgendorfi / Scorpaeniformes	Yes			Ueck & Kobayashi 1979.
Monopterus chuchia / Scorpaeniformes			Yes	Sastry & Sathyanesan 1981.
Platichthys flesus / Pleuronectiformes		Yes		Borg, Ekstrom & Van Veen 1983.
Solea senegalensis / Pleuronectiformes	Yes			Confente 2018.

Supplementary Table 2: Neurochemical markers analysed in the parapineal of zebrafish at larval and adult stages.

Neurochemical Marker	7dpf	Adult
Serotonin (5HT)	+	+
Substance P (SP)	+	+
Tachykinin (TAC: SP precursor)	+	+
Neuropeptide Y (NPY)	-	-
Gad 65/67 (GABA)	-	-
Choline acetyltransferase (ChAT)	-	-
Tyrosine Hydroxylase (TH)	-	-

Supplementary Figure 1

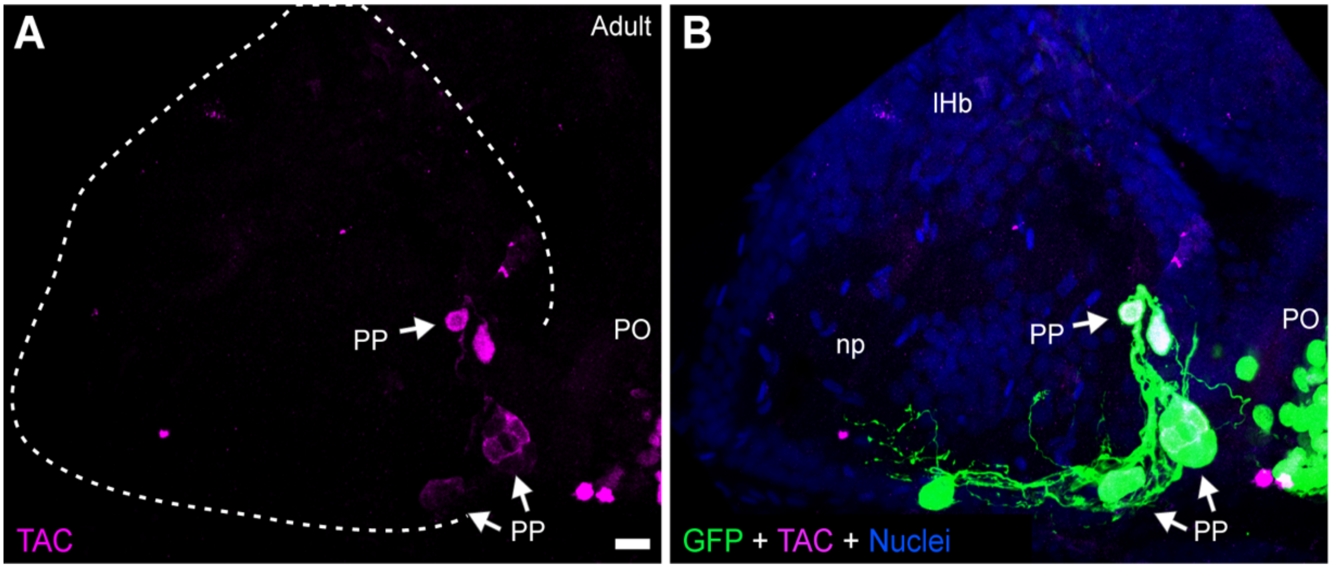

Supplementary Figure 1: Parapineal cells show immunoreactivity against tachykinin, the precursor of substance P. Immunofluorescence against tachykinin (Tac) in adult Tg(*foxd3::GFP*) zebrafish. Images correspond to confocal z-stack maximum projections showing the fluorescence signal corresponding to Tac (magenta in **A**), or the merge fluorescence signals that also include the GFP of the pineal complex (green) and the DAPI/Hoechst nuclear staining that provides the left habenula tissue context (blue) (**B**). White arrows indicate Tac-immunoreactive parapineal cell bodies. Abbreviations: PO (pineal organ at the level of the stalk), PP (parapineal). Adult samples (n=2). Scale bar, 10µm.

Supplementary References

1. Birba, A., Ramallo, M. R., Morandini, L., Villafañe, V., Tubert, C., Guimarães Moreira, R., & Pandolfi, M. (2014). The pineal complex in the cichlid *Cichlasoma dimerus*: effect of different photoperiods on its cell morphology. *Journal of Fish Biology*, *85*(3), 605-620.
2. Borg, B., Ekström, P., & van Veen, T. (1983). The parapineal organ of teleosts. *Acta Zoologica*, *64*(4), 211-218.
3. Confente, F., El M'Rabet, A., Ouarour, A., Voisin, P., De Grip, W. J., Rendón, M. C., & Muñoz-Cueto, J. A. (2008). The pineal complex of Senegalese sole (*Solea senegalensis*): Anatomical, histological and immunohistochemical study. *Aquaculture*, **285**(1–4), 207– 215.
4. Dey, R., Bhattacharya, S., Maitra, S. K., & Banerji, T. K. (2003). The morpho-anatomy and histology of the pineal complex in a major indian carp, *Catla catla*: Identification of the pineal photoreceptor cells and their responsiveness to constant light and constant darkness during different phases of the annual reproductive cycle. *Endocrine research*, *29*(4), 429-443.
5. Ishikawa, Y., Inohaya, K., Yamamoto, N., Maruyama, K., Yoshimoto, M., Iigo, M., ... & Ito, H. (2015). The parapineal is incorporated into the habenula during ontogenesis in the medaka fish. *Brain, Behavior and Evolution*, *85*(4), 257-270.
6. Herrera-Pérez, P., Servili, A., Rendón, M. C., Sanchez-Vazquez, F. J., Falcon, J., & Munoz-Cueto, J. A. (2011). The pineal complex of the European sea bass (*Dicentrarchus labrax*): I. Histological, immunohistochemical and qPCR study. *Journal of Chemical Neuroanatomy*, *41*(3), 170-180.
7. Hill, C. (1894). *The epiphysis of teleosts and Amia.* Reprinted from Journal of Morphology, IX(2). Ginn & Company. Boston.
8. Holmgren, U. (1965). On the ontogeny of the pineal and parapineal organs in teleost fishes. *Structure and Function of the Epiphysis Cerebri*, *10*, 172.
9. McNulty, J. A. (1976). A comparative study of the pineal complex in the deep-sea fishes *Bathylagus wesethi* and *Nezumia liolepis*. *Cell and Tissue Research*, *172*(2), 205-225.
10. McNulty, J. A., & Nafpaktitis, B. G. (1977). Morphology of the pineal complex in seven species of lanternfishes (Pisces: Myctophidae). *American Journal of Anatomy*, *150*(4), 509-529.
11. McNulty, J. A. (1978). A light and electron microscopic study of the pineal in the blind goby, *Typhlogobius californiensis* (Pisces: Gobiidae). *Journal of Comparative Neurology*, *181*(1), 197-211.
12. Musæus, F. J. (2009). The pineal gland of Arctic charr (*Salvelinus alpinus L.*): a survey of seasonal melatonin production and gland ultrastructure (Master's thesis, Universitetet i Tromsø).
13. Örği, E., & Oğuz, A. R. (2022). Anatomical and histological investigation of the pineal gland in the lake van fish (*Alburnus tarichi* (Güldenstädt, 1814)). *Anatomia, Histologia, Embryologia*, 51, 427– 434.
14. Rincón Camacho, L., Morandini, L., Birba, A., Cavallino, L., Alonso, F., LoNostro, F. L., & Pandolfi, M. (2016). The pineal complex: a morphological and immunohistochemical comparison between a tropical (*Paracheirodon axelrodi*) and a subtropical (*Aphyocharax anisitsi*) characid species. *Journal of Morphology*, *277*(10), 1355-1367.
15. Rüdeberg, C. (1968). Structure of the parapineal organ of the adult rainbow trout, *Salmo gairdneri* Richardson. *Zeitschrift für Zellforschung und Mikroskopische Anatomie*, *93*(2), 282-304.
16. Rüdeberg, C. (1969). Light and electron microscopic studies on the pineal organ of the dogfish, *Scyliorhinus canicula L. Zeitschrift für Zellforschung und mikroskopische Anatomie*, *96*(4), 548-581.
17. Rüdeberg, C. (1971). Structure of the pineal organs of Anguilla *anguilla L.* and *Lebistes reticulatus* Peters (Teleostei). *Zeitschrift für Zellforschung und Mikroskopische Anatomie*, *122*(2), 227-243.
18. Sastry, V. K., & Sathyanesan, A. G. (1981). A comparative study of the pineal complex of nineteen species of Indian freshwater fishes. *Journal fur Hirnforschung*, *22*(3), 327-340.

19. Shrivastava RK. (1972). Studies on the Functional Morphology and Anatomy of the Central Nervous System of *Xenentodon cancila* (Ham.) including its Histology 1. The Brain. *Okajimas Folia Anatomica Japonica*, *49*(2-3), 111-127.
20. Ueck, M., & Kobayashi, H. (1978). Vergleichende Untersuchungen am Pinealorgan von Fischen des Pazifischen Ozeans. Abstract of *6th Deutsche Neurobiologentagung, Göttingen.*
21. Ueck, M. (1979). Innervation of the vertebrate pineal. *Progress in Brain Research*, *52*, 45-88.
22. Van Veen, T., Ekström, P., Borg, B., & Møller, M. (1980). The pineal complex of the three-spined stickleback, *Gasterosteus aculeatus L*. *Cell and tissue research*, *209*(1), 11-28.
23. Vigh-Teichmann, I., Korf, H. W., Oksche, A., & Vigh, B. (1982). Opsin-immunoreactive outer segments and acetylcholinesterase-positive neurons in the pineal complex of *Phoxinus phoxinus* (Teleostei, Cyprinidae). *Cell and tissue research*, *227*(2), 351-369.
24. Vigh-Teichmann, I., Szél, A., Röhlich, P., & Vigh, B. (1990). A comparison of the ultrastructure and opsin immunocytochemistry of the pineal organ and retina of the deep-sea fish *Chimaera monstrosa*. *Experimental biology*, *48*(6), 361-371.
25. Vigh-Teichmann, I., Ali, M. A., Szél, A., & Vigh, B. (1991). Ultrastructure and opsin immunocytochemistry of the pineal complex of the larval Arctic charr *Salvelinus alpinus*: a comparison with the retina. *Journal of pineal research*, *10*(4), 196-209.
26. Yañez, J., & Anadón, R. (1996). Afferent and efferent connections of the habenula in the rainbow trout (*Oncorhynchus mykiss*): an indocarbocyanine dye (DiI) study. *Journal of Comparative Neurology*, *372*(4), 529-543.
